# Incidence, Risk Factors, and Clinical Outcomes of Acute Kidney Injury Caused by Palliative Chemotherapy in Lung Cancer

**DOI:** 10.7150/jca.28399

**Published:** 2019-08-29

**Authors:** Song Ee Park, Jin Ho Hwang, Jin Hwa Choi, Su-Hyun Kim, Jae Chol Choi, Joung Soon Jang, Hee Jun Kim, Suk Won Park, Ju Won Seok, In Gyu Hwang

**Affiliations:** 1 Division of Hemato-oncology, Department of Internal Medicine, Chung-Ang University College of Medicine, Seoul, Korea; 2 Division of Nephrology, Department of Internal Medicine, Chung-Ang University College of Medicine, Seoul, Korea; 3 Department of Radiation Oncology, Chung-Ang University College of Medicine, Seoul, Korea; 4 Division of Pulmonology, Department of Internal Medicine, Chung-Ang University College of Medicine, Seoul, Korea.; 5 Department of Radiation Oncology, Myungji Hospital, Goyang, Korea; 6 Department of Nuclear Medicine, Chung-Ang University College of Medicine, Seoul, Korea.; Song Ee Park and Jin Ho Hwang Contributed equally to this work.

**Keywords:** Acute kidney injury (AKI), Lung cancer, Chemotherapy, Incidence, Survival, Risk factor

## Abstract

**Purpose**: Acute kidney injury (AKI) affects cancer therapy outcome and increases morbidity and mortality in cancer patients. We investigated the incidence, risk factors, and clinical outcomes of AKI caused by palliative chemotherapy in lung cancer patients.

**Materials and Methods**: Between January 2005 and November 2014, 207 lung cancer patients who had been treated with first-line palliative chemotherapy were enrolled. Renal function was assessed during every cycle of chemotherapy. AKI was defined based on changes in serum creatinine levels as described in the Kidney Disease: Improving Global Outcomes guidelines. Clinical outcomes were evaluated depending on AKI occurrence during the first-line chemotherapy.

**Results**: Of the 207 patients, 36 (17.4%) experienced AKI. Among the 36 patients who developed AKI during chemotherapy, 33 (91.8%) had AKI stage I. Although 19 patients (52.7%) with AKI during chemotherapy progressed to chronic kidney disease (CKD), no patients were reported to progress to end-stage renal disease (ESRD). The number of chemotherapy cycles was independently associated with chemotherapy-induced AKI in multivariate analysis (OR = 1.71, 95% CI 1.29-2.26, p < 0.001). The median follow-up duration was 83 months. Patients with AKI during chemotherapy (AKI group) showed significantly longer time to treatment failure than patients without AKI (non-AKI group) (4.2 vs. 2.5 months, p < 0.001). However, the median overall survival (11.7 vs. 8.8 months, p = 0.147) and progression-free survival (5.5 vs. 5.2 months, p = 0.347) were not different between the groups.

**Conclusions**: AKI that developed during chemotherapy was mostly of mild degree and its prognosis was favorable. The occurrence of AKI was associated with the number of chemotherapy cycles administered. AKI did not adversely affect survival of lung cancer patients during chemotherapy.

## Introduction

Acute kidney injury (AKI) is a commonly encountered complication in cancer patients, and it can interrupt cancer therapy and increase patient morbidity and mortality 1. Cancer patients may be at increased risk of AKI owing to malignant infiltration, tumor lysis syndrome, urinary tract obstruction, sepsis, radiotherapy, or use of nephrotoxic drugs 2, 3. In addition, common comorbidities to cancer such as chronic kidney disease, congestive heart failure, hypertension, diabetes, and liver disease may increase the risk of AKI 4-6. Furthermore, cancer therapy is becoming more common in elderly patients, a subpopulation that is particularly vulnerable to the adverse nephrotoxic effects of many drugs as well as to the use of intravenous radiocontrast agents 7. AKI can preclude optimal management of malignancy as changes in chemotherapeutic drug pharmacokinetics related to renal dysfunction may result in under-dosing, with a decreased chance of achieving remission, or in over-dosing, with an increased risk of toxicity 8. There are several direct or indirect risk factors for AKI development during palliative chemotherapy in advanced non-small cell lung cancer (NSCLC) and extensive-stage small cell lung cancer (SCLC). The reference chemotherapeutic regimen for lung cancer is a double combination of a platinum compound (cisplatin or carboplatin) with gemcitabine, vinorelbine, or taxanes (paclitaxel or docetaxel) 9, and AKI occurs in 20%-30% of patients treated with these drugs 10. Risk factors that are known to increase the risk of cisplatin-induced nephrotoxicity include genetic variation, race, sex, age, comorbidity, and smoking 11.

To date, although there have been several studies on the incidence and risk factors of AKI in cancer patients, there has been no previous study analyzing the incidence of AKI and its impact on clinical outcomes in lung cancer patients treated with chemotherapy. This study aimed to investigate the incidence and risk factors of AKI in lung cancer patients during first-line palliative chemotherapy and to evaluate the effects of AKI on prognosis and treatment outcomes.

## Materials and Methods

### Study Design and Patients

This is retrospective cohort study of adult patients who had undergone first-line palliative chemotherapy for the treatment of lung cancer from January 2005 to November 2014 at Chung-Ang University Hospital, Seoul, South Korea. A total of 255 patients were reviewed and 207 were enrolled in the study.

The inclusion criteria were as follows: (1) diagnosis of advanced non-small cell lung cancer (NSCLC, stage IIIB-stage IV) or extensive-stage small cell lung cancer (SCLC ED); SCLC ED means stage IV (T any, N any, M1a/b) or T3-4 cancer caused by multiple lung nodules that are too extensive or have tumor/nodal volume that is too large to be encompassed in a tolerable radiation plan 12; (2) administration of first-line palliative chemotherapy; and (3) availability of baseline serum creatinine values. Patients were excluded if they (1) received epidermal growth factor receptor tyrosine kinase inhibitors (gefitinib, erlotinib) and anaplastic lymphoma kinase inhibitor (crizotinib); (2) received concurrent chemoradiotherapy or chemotherapy followed by radiotherapy; (3) had an Eastern Cooperative Oncology Group (ECOG) score ≥ 3; or (4) had received previous adjuvant chemotherapy within the past 6 months.

### Estimation of Glomerular Filtration Rate and Definition of Acute Kidney Injury

The estimated glomerular filtration rate (eGFR, mL/min/1.73 m^2^) was calculated using the Chronic Kidney Disease Epidemiology (CKD-EPI) equation 13, 14. eGFR was evaluated before initiating each palliative chemotherapy cycle, after the last chemotherapy cycle, and every 6 months during the follow-up period. AKI was defined based on changes in serum creatinine (SCr) levels as described in the Kidney Disease: Improving Global Outcomes (KDIGO) guidelines. AKI was defined as an absolute increase in SCr level by ≥0.3 mg/dL within 48 hours, and/or an increase of more than 1.5-fold from the baseline SCr level within 7 days. The severity of AKI was classified into three stages as follows: AKI stage 1, a 1.5-1.9-fold increase in SCr levels from baseline or an increase by ≥0.3 mg/dL within 48 hours; AKI stage 2, a 2.0-2.9-fold increase in SCr levels from baseline; and AKI stage 3, >3-fold increase in SCr levels from baseline or SCr level of ≥4.0 mg/dL or initiation of renal replacement therapy 15. In this study, urine output criteria were not considered because of the inaccuracy of the data being collected retrospectively. Complete recovery was defined as the absence of AKI, and incomplete recovery as persistent AKI with a decrease in AKI stage compared with their maximal AKI stage. A persistent increase in absolute SCr of ≥0.3 mg/dL or ≥1.5-fold from the baseline SCr level was also considered as incomplete recovery 15. For each AKI event, possible causes or associated factors were retrospectively evaluated. Renal outcomes after the events were divided as follows: complete recovery from the AKI; progression to chronic kidney disease (CKD); end-stage renal disease (ESRD); or death with functioning kidney. AKI occurrence was evaluated during first-line palliative chemotherapy.

### Other Definitions and Grouping

Relative dose intensity (RDI) was defined as the ratio of the delivered dose of a single drug (or of several drugs in a combination chemotherapy regimen) to the planned dose of the drug 8. Time to treatment failure (TTF) was defined as the duration from the first day of first-line palliative chemotherapy to discontinuation of treatment for any reason, including disease progression, treatment toxicity, patient's refusal, or death. Progression-free survival (PFS) was the duration from the first day of first-line palliative chemotherapy to disease progression or death. Overall survival (OS) was defined as duration from the first day of first-line palliative chemotherapy to death from any cause. Early cycle was defined as within two cycles of chemotherapy, late cycle was defined as three or more cycles of chemotherapy. To identify the risk factors potentially associated with the occurrence of AKI during chemotherapy, we examined the relationship between AKI incidence and the following CKD risk factors: old age (≥70 years), diabetes mellitus, hypertension, and smoking 16. We also assessed the following potential risk factors: sex, ECOG 0-1 vs. 2, cisplatin use, and the number of chemotherapy cycles conducted 17-19. We accepted general management for AKI prevention. To increase the renal excretion of cisplatin, fluid therapy was uniformly conducted with normal saline. Normal saline was administered at a rate of 500 mL/hr and separately 1 L normal saline before and after cisplatin, and simultaneously 20% mannitol 70 mL was administered after cisplatin administration 20, 21.

### Outcome Measurements

We evaluated AKI incidence during first-line palliative chemotherapy, TTF, PFS, OS, risk factors, and therapeutic compliance according to the occurrence of AKI. Each end point was measured at the beginning of every chemotherapy cycle. The objective response rate (ORR) was assessed by the Response Evaluation Criteria In Solid Tumors (RECIST) 1.1.

### Statistical Analysis

All statistical analyses were performed using the SPSS 19 (SPSS Inc., Chicago, IL, USA) software package. Differences in categorical variables between the two groups were determined by Chi-square test. Differences in continuous variables between the two groups were compared using Student's *t*-test. Categorical data are expressed as percentages, and continuous variables are expressed as means ± standard deviation or median (range). For all tests, a P value (two-tailed) of <0.05 was considered statistically significant.

The logistic regression model was used to identify independent risk factors of AKI by calculating odds ratios (ORs) and 95% confidence intervals (CIs). Univariate analyses were performed with variables, such age (<70 of age or ≥70 years of age), sex, cancer stage, performance status, response, smoking, co-morbidity, and cisplatin use. In addition, multivariate analysis was conducted using logistic regression models for variables with a P value <0.1 in the univariate analysis including achievement of response, chemotherapy cycle, and smoking.

The Institutional Review Board of the Chung-Ang University College of Medicine (C2015003) approved the study. The requirement of informed consent was waived as the study was based on the retrospective analyses of existing administrative and clinical data.

## Results

### Patient characteristics

A total of 255 patients with lung cancer were reviewed and 207 were finally included in this study (Fig. S1). There were 168 (81.2%) male patients and the median age of all the patients was 68 years (range, 33-84 years). One hundred and ninety-three (93.2%) patients had a ECOG score of 0-1; 28 (13.5%) had NSCLC stage IIIB, 113 (54.6%) had NSCLC stage IV, and 66 (31.9%) had SCLC ED. Almost half of the patients were smokers (48.6%), whereas only four subjects had prior renal disease (1.9%). The patients received various cisplatin- or carboplatin-based combination regimens with etoposide (26.6%), gemcitabine (26.1%), paclitaxel (23.2%), docetaxel (9.7%), pemetrexed (8.2%), irinotecan (5.3%), and others (1.0%) (Table 1).

### Incidence of AKI

**Fig 1:**
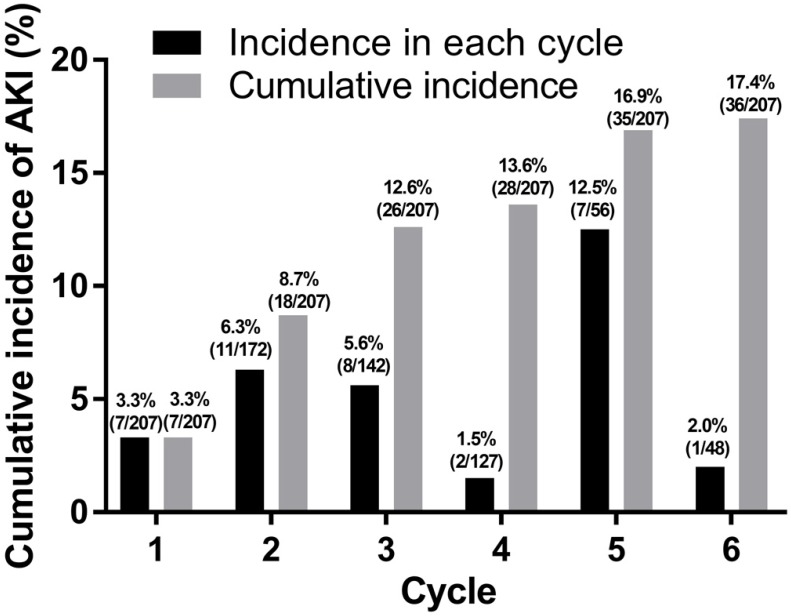
Incidence of acute kidney injury.

From a total of 207 patients, 36 (17.4%) patients experienced 38 episodes of AKI during first-line palliative chemotherapy (Fig. 1). Among these 36 patients, 16 (44.4%) experienced AKI at an age of 70 years or older. There was no significant difference in the occurrence of AKI during chemotherapy between patients aged ≥70 years and those aged <70 years (P = 0.514).

### Risk factors for developing AKI

Results of the univariate and multivariate analyses of risk factors for AKI during chemotherapy are summarized in Table 2. In univariate analysis, achievement of response (OR = 3.90, 95% CI 1.82-8.35, P < 0.001) and the number of chemotherapy cycles administered (chemotherapy cycle) (OR = 1.61, 95% CI 1.30-1.98, P < 0.001) were identified as significant risk factors for AKI during chemotherapy. Age (≥ 70) (OR = 1.27, 95% CI 0.62-2.63, p = 0.515), diabetes mellitus (OR = 1.35, 95% CI 0.60-3.04, p = 0.474), hypertension (OR = 1.17, 95% CI 0.57-2.41, p = 0.665), previous renal disease (OR = 4.97, 95% CI 0.68-36.52, p = 0.115) and cisplatin (OR = 3.05, 95% CI 0.69-13.47, p = 0.142) did not affect the occurrence of chemotherapy-induced AKI. The multivariable analysis showed the number of chemotherapy cycles was independently associated with chemotherapy-induced AKI (OR = 1.39, 95% CI 1.13-1.71, p = 0.001) (Table 2).

### Clinical Outcomes and AKI

The 207 patients with lung cancer received a total of 759 cycles of the first-line palliative chemotherapy during the study period. The median number of chemotherapy cycle was 4 (range: 1 - 14). Fourteen patients received more than 6 cycles of treatment. The median number of the conducted chemotherapy cycles was different between the AKI group and the non-AKI group (5 vs. 4 cycles, range 1-14 vs. 1-12, p < 0.001) (Table 3). Six (2.9%) patients showed complete response (CR) and 76 (36.7%) patients showed partial response (PR). More favorable ORR to chemotherapy was shown in the AKI group (34.0% vs. 66.6%, p < 0.001). Disease control rate (DCR) with CR, PR, and SD was 83.3% in the AKI group (p = 0.001) (Table 3).

None of the 36 patients with AKI required chemotherapy dose reduction or chemotherapy delay due to AKI. Among the patients with AKI, chemotherapy was discontinued due to disease progression in 25 patients and toxicity in 6 patients. The causes of toxicity were neutropenia, arrhythmia, pneumonia, anorexia, and cerebral infarction. Additionally, one patient required a change in regimen from cisplatin to carboplatin because of the development of AKI.

**Fig 2:**
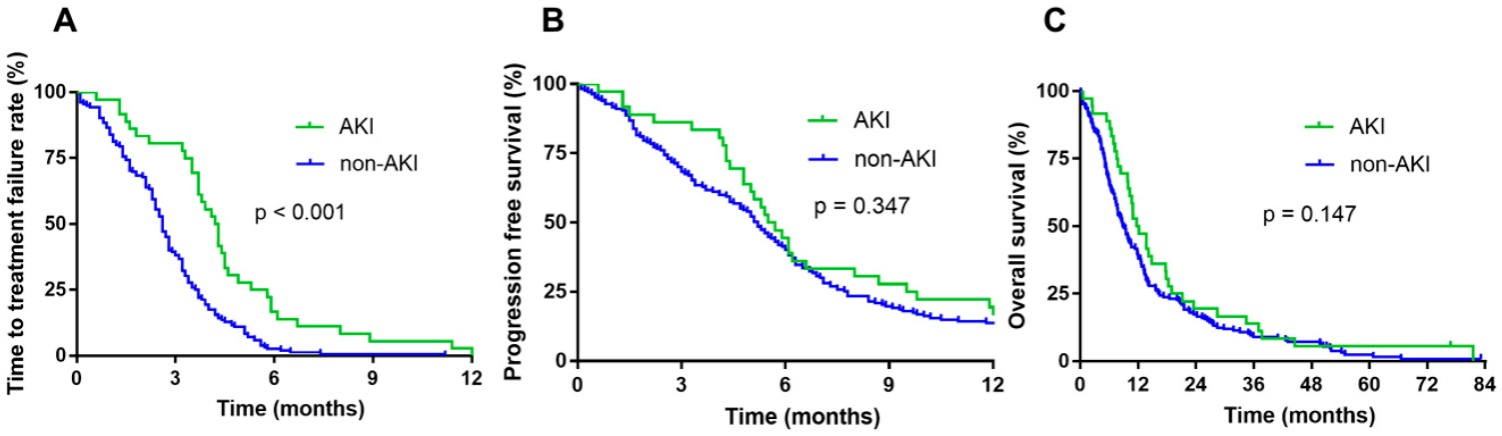
** Survival according to renal function during chemotherapy.** Time to treatment failure (TTF) (A), progression-free survival (PFS) (B), and overall survival (OS) of patients with AKI during chemotherapy (C).

The cutoff date for analyses was April 2018, resulting in a median follow-up duration of 83 months. The occurrence of AKI during chemotherapy did not affect the median OS (11.7 vs. 8.8 months, p = 0.147) and PFS (5.5 vs. 5.2 months, p = 0.347). However, the AKI group showed significantly longer TTF than the non-AKI group (4.2 vs. 2.5 months, p < 0.001) (Fig. 2).

### Renal Outcomes of AKI during Chemotherapy

During chemotherapy, most patients with AKI (92.1%) showed AKI stage I, and 42.1% of the patients recovered fully to baseline renal function. Platinum chemotherapeutic agents including cisplatin were the most common cause of AKI during chemotherapy, affecting 24 patients (66.7%). Among patients with AKI induced by platinum chemotherapy, 8 patients (33.4%) showed complete recovery from AKI, AKI progressed to CKD in 15 patients (62.5%), and no patient progressed to end-stage renal disease (ESRD). The second most common cause of AKI during chemotherapy was contrast-induced nephropathy (CIN), which was responsible for AKI in 5 patients; however, it was associated with a favorable outcome (4 patients (80.0%) showed complete recovery from AKI). Among patients who developed AKI during chemotherapy of any cause, 19 patients (52.7%) progressed to CKD. Among patients who developed AKI during chemotherapy, 2 patients with AKI recurrence developed respectively CIN and chemotherapy induced AKI while undergoing chemotherapy. The other types of AKI during chemotherapy were prerenal AKI (4 patients), septic AKI (1 patient), and postrenal AKI (1 patient).

## Discussion

In this study, we determined the incidence, risk factors, and prognosis of AKI caused by first line palliative chemotherapy in patients with lung cancer according to the recently published and validated KDIGO guidelines. Although previous studies have reported less toxic methods for chemotherapy administration, such as normal saline infusion with supplementation of magnesium and mannitol, chemotherapy-induced AKI still occurs in 6-14% of patients 22, 23; the incidence of AKI during chemotherapy was similar in our study. In this study, AKI incidence during the first-line chemotherapy revealed three characteristics: First, the risk of AKI during chemotherapy increased with an increase in the number of chemotherapy cycles in multivariable analysis. Second, there was no significant difference in AKI incidences between the elderly and younger patients. Third, the most common cause of AKI during chemotherapy was the platinum chemotherapeutic agent.

Previous studies have demonstrated that old age (≥70 years), female sex, current smoking, cardiovascular diseases (hypertension and ischemic heart disease), and diabetes mellitus increase the risk of AKI in lung cancer patients 18, 22, 24. In this study, the occurrence of AKI was only associated with the number of chemotherapy cycles administered. Age, combination chemotherapy, and comorbidities did not affect the occurrence of AKI during chemotherapy. Most of the elderly patients developed mild AKI, but continued receiving chemotherapy.

A previous study reported that a cumulative exposure to cisplatin can cause acute tubular necrosis and may lead to glomerular damage 5. In another study, nephrotoxicity developed in 28-36% patients who received a single dose (50 mg/m^2^) of cisplatin 25. In our study, nephrotoxicity was found in 18.9% of patients who received cisplatin-based combination chemotherapy; moreover, the odds ratio of developing AKI increased by 1.61 times with an increase in a single cycle of chemotherapy.

TTF was significantly longer in the AKI group than in the non-AKI group; however, PFS and OS was not different between the two groups. The reason for the discrepancy between TTF and PFS is likely that AKI that developed in the early cycles of the first-line chemotherapy was mild and reversible, and the chemotherapy could be continued in most cases; conversely, AKI developed in the late cycles of the first-line chemotherapy was caused by long-term chemotherapy. Furthermore, these patients had better ORR and DCR than those in the non-AKI group. This result may imply that treatment-induced AKI is associated with properly conducted chemotherapy and favorable outcomes in lung cancer patients. Another possible reason is that the non-AKI group had a higher number of patients refusing treatment. Therefore, the duration of AKI development did not affect survival.

AKI that developed during chemotherapy did not affect survival, and it was not determined on the basis of the AKI grade. In our study, prognosis of AKI during chemotherapy was favorable as none of the patients with AKI developed ESRD. If AKI develops during chemotherapy, we suggest careful continuation of the chemotherapy for better prognosis.

We assessed the occurrence of AKI according to the KDIGO AKI definition. This could have been the cause of disparity between the retrospective AKI diagnosis and detection of AKI at that time point by the physician. There was a tendency of delayed AKI detection, especially for mild AKI (Stage 1). Patients who developed stage 1 AKI were usually prescribed the same chemotherapeutic agents with the same doses until more severe AKI or persistent renal function deterioration developed. Occurrence of stage 1 AKI with normal renal function could remain easily undetected, especially when the increased serum creatinine level was also within the normal range. It could be also related to the positive outcome of TTF in the AKI group. The difference between PFS and TTF in the non-AKI group was attributable to other causes except disease progression. The most common cause of discontinued chemotherapy in the non-AKI group was patient's refusal (33 patients). Eighteen patients discontinued chemotherapy due to toxicity from causes other than AKI, and 13 patients discontinued chemotherapy due to physician reluctance. For the above reasons, the response could not be evaluated in 20.5% patients in the non-AKI group.

This study had some limitations. The study has a retrospective design, associations could only be inferred. There could be inevitable biases due to patient selection, follow-up, or interpretation of the outcomes. The AKI group received more cycles of chemotherapy because the AKI events mostly were mild and reversible or possibly because compliance of the AKI group was better than that of the others. Therefore, we concluded that physicians should be more careful about assessing the AKI events. Despite the limitations, we strictly defined AKI according to the latest guidelines. In addition, our study examined the relation between AKI and its risk factors, clinical outcomes, and prognosis during chemotherapy. Our results are meaningful because we focused on the occurrence and prognosis of AKI itself as well as that of cancer-related outcomes.

AKI that developed during chemotherapy in lung cancer patients was mostly mild and associated with the number of chemotherapy cycles administered. The prognosis of AKI during chemotherapy was favorable, and it did not progress to ESRD. Further, AKI occurrence in such patients did not influence survival. Therefore, appropriate treatment of AKI should be performed and the continuation of chemotherapy should be determined.

## Supplementary Material

Supplementary figure and table.

## Figures and Tables

**Table 1:** Baseline clinical characteristics during chemotherapy.

	Total (N=207)	No AKI during CTx (n=171)	AKI during CTx (n=36)	*p* value
Median age, years (range)	68 (33-84)	68 (33-84)	66 (47-81)	0.741
Age ≥ 70 years	82 (39.6)	66 (38.6)	16 (44.4)	0.514
Male	168 (81.2)	139 (81.3)	29 (80.6)	0.919
Body weight (kg)	59.5 ± 12.3	59.7 ± 12.7	58.6 ± 10.1	0.637
Height (cm)	161.4 ± 17.7	161.2 ± 19.0	162.2 ± 7.3	0.774
BSA (m^2^)	1.61 ± 0.28	1.62 ± 0.27	1.57 ± 0.33	0.384
Smoking status	100 (48.6)	88 (51.5)	12 (34.3)	0.048
Smoking (pack-years)	22.3 ± 42.3	24.4 ± 45.0	12.5 ± 20.2	0.130
Comorbidity				
DM	48 (23.2)	38 (22.2)	10 (27.8)	0.474
HT	91 (44.0)	74 (43.3)	17 (47.2)	0.665
Pulmonary disease	24 (11.6)	22 (12.9)	2 (5.6)	0.213
Cardiac disease	19 (9.2)	16 (9.4)	3 (8.3)	0.847
CKD (by history-taking)^*^	4 (1.9)	2 (1.2)	2 (5.6)	0.082
CKD (by eGFR)^a^	21 (10.1)	19 (11.1)	2 (5.6)	0.539
ECOG				
0-1	193 (93.2)	160 (93.6)	33 (91.7)	0.680
2	14 (6.8)	11 (6.4)	3 (8.3)	
Histology of NSCLC				
Adenocarcinoma	87 (42.0)	72 (42.1)	15 (41.7)	0.925
Squamous CC	51 (24.6)	43 (25.1)	8 (22.2)	
Large CC	1 (0.5)	1 (0.6)	0 (0)	
Small CC	65 (31.4)	53 (31.0)	12 (33.3)	
Others	3 (1.4)	2 (1.2)	1 (2.8)	
Stage				
NSCLC IIIB	28 (13.5)	23 (13.5)	5 (13.9)	0.971
NSCLC IV	113 (54.6)	94 (55.0)	19 (52.8)	
SCLC ED	66 (31.9)	54 (31.5)	11 (33.3)	
Combination CTx				
Etoposide	55 (26.6)	45 (26.3)	10 (27.8)	0.972
Gemcitabine	54 (26.1)	46 (26.9)	8 (22.2)	
Paclitaxel	48 (23.2)	39 (22.8)	9 (25.0)	
Docetaxel	20 (9.7)	17 (9.9)	3 (8.3)	
Pemetrexed	17 (8.2)	13 (7.6)	4 (11.1)	
Irinotecan	11 (5.3)	9 (5.3)	2 (5.6)	
Other	2 (1.0)	2 (1.2)	0 (0)	
Cisplatin use	179 (86.5)	145 (84.8)	34 (94.4)	0.124
Carboplatin use	22 (10.6)	20 (11.7)	2 (5.6)	0.277

AKI, acute kidney injury; AKI during CTx, occurrence of AKI during the first line chemotherapy; No AKI during CTx, without AKI during the first line chemotherapy; BSA, body surface area; DM, diabetes mellitus; HTN, hypertension; CKD, chronic kidney disease; eGFR, estimated glomerular filtration rate; ECOG, eastern cooperative oncology group performance status; NSCLC, non-small cell lung cancer; SCLC ED, extensive stage small cell lung cancer; Squamous CC, squamous cell carcinoma; Large CC, large cell carcinoma; Small CC, small cell carcinoma; CTx, chemotherapy. ^a^CKD by history taking is based on patients' awareness of having CKD, and CKD by eGFR is based on the calculated CKD-EPI eGFR < 60 ml/min/1.73 m^2^.

**Table 2:** Univariate and multivariate analyses of factors associated with AKI incidence during chemotherapy (N=207).

	Univariate analysis			Multivariate analysis		
Variables	Odds ratio	95% CI	*p* value	Odds ratio	95% CI	*p* value
Age > 70 years	1.27	0.62-2.63	0.515			
Female	1.05	0.42-2.61	0.919			
Stage						
NSCLC III	Reference					
NSCLC IV	0.93	0.31-2.75	0.895			
SCLC ED	1.02	0.32-3.23	0.970			
Histology						
SCLC	1.08	0.50-2.33	0.837			
ECOG 2	1.32	0.35-5.00	0.681			
ORR of SD or PD	3.90	1.82-8.35	<0.001	1.83	0.75-4.44	0.181
Chemotherapy cycle^a^	1.61	1.30-1.98	<0.001	1.39	1.13-1.71	0.001
Smoking	0.47	0.22-1.00	0.051	0.54	0.24-1.21	0.135
DM	1.35	0.60-3.04	0.474			
HTN	1.17	0.57-2.41	0.665			
Pulmonary disease	0.40	0.09-1.78	0.228			
Cardiac disease	0.88	0.24-3.20	0.847			
CKD	4.97	0.68-36.52	0.115			
Cisplatin	3.05	0.69-13.47	0.142			

CI, confidence interval; NSCLC, non-small cell lung cancer; SCLC ED, extensive stage small cell lung cancer; DM, diabetes mellitus; HTN, hypertension; ORR, objective response rate; AKI, acute kidney injury. ^a^The number of cycles of chemotherapy administered.

**Table 3:** Response according to renal function during chemotherapy and chemotherapy compliance (N=207).

Efficacy of CTx	Total (N=207)	No AKI during CTx (n=171)	AKI during CTx (n=36)	*p* value
Chemotherapy response				
CR, n (%)	6 (2.9)	3 (1.8)	3 (8.3)	0.002
PR	76 (36.7)	55 (32.2)	21 (58.3)	
SD	43 (20.8)	37 (21.8)	6 (16.7)	
PD	46 (22.2)	41 (24.0)	5 (13.9)	
not evaluable	36 (17.4)	35 (20.5)	1 (2.8)	
ORR	82 (39.6)	58 (34.0)	24 (66.6)	<0.001
DCR	125 (60.4)	95 (55.8)	30 (83.3)	0.001
Median cycle, range	4 (1-14)	4 (1-12)	5 (1-14)	<0.001
RDI	99.05 ± 5.6	98.9 ± 6.0	99.5 ± 2.5	0.530

CTx, chemotherapy; AKI, acute kidney injury; AKI during CTx, occurrence of AKI during the first line chemotherapy; No AKI during CTx, without AKI during the first line chemotherapy; CR, complete response; PR, partial response; SD, stable disease; PD, progressive disease; ORR, objective response rate; DCR, disease control rate.

## Abbreviations

AKI: acute kidney injury
CKD: chronic kidney disease
eGFR: estimated glomerular filtration rate
ESRD: end-stage renal disease
NSCLC: non-small cell lung cancer
ORR: overall response rate
OS: overall survival
PFS: progression-free survival
SCLC: small cell lung cancer
TTF: time to treatment failure
