# Supplementary material for: Incidence, Risk Factors, and Clinical Outcomes of Acute Kidney Injury Caused by Palliative Chemotherapy in Lung Cancer
Source: J Cancer. 2019 Aug 29;10(22):5332–8. doi: 10.7150/jca.28399 (PMC6775698; doi:10.7150/jca.28399)

Table S1. Stage and clinical course of AKI during chemotherapy (n=36 patients).

(**A**) Stage of AKI during chemotherapy (n=36)

	AKI stage I	AKI stage II	AKI stage III
**Episodes of AKI**	33 (91.8 %)	1 (2.7%)	2 (5.5%)

(**B**) Clinical course of AKI during chemotherapy

**AKI during chemotherapy**		**Clinical course, number of patients (%)**			
**Cause**	**Patients (n=36)**	**Complete recovery from AKI (n=16)**	**AKI progress to CKD (n=19)**	**ESRD (n=0)**	**Death with functioning kidney (n=1)**
**Platinum**	24	8 (33.4)	15(62.5)	0	1 (4.1)
**Contrast-induced nephropathy (CIN)**	5	4 (80.0)	1 (20.0)	0	0
**Volume depletion**	4	2 (50.0)	2 (50.0)	0	0
**Other toxic AKI**	1	0	1 (100.0)	0	0
**Septic AKI**	1	1 (100.0)	0	0	0
**Post-renal AKI**	1	1 (100.0)	0	0	0

AKI, acute kidney injury; CKD, chronic kidney disease; ESRD, end-stage renal disease.

Fig S1. Profile of patients with lung cancer who received first-line palliative chemotherapy.

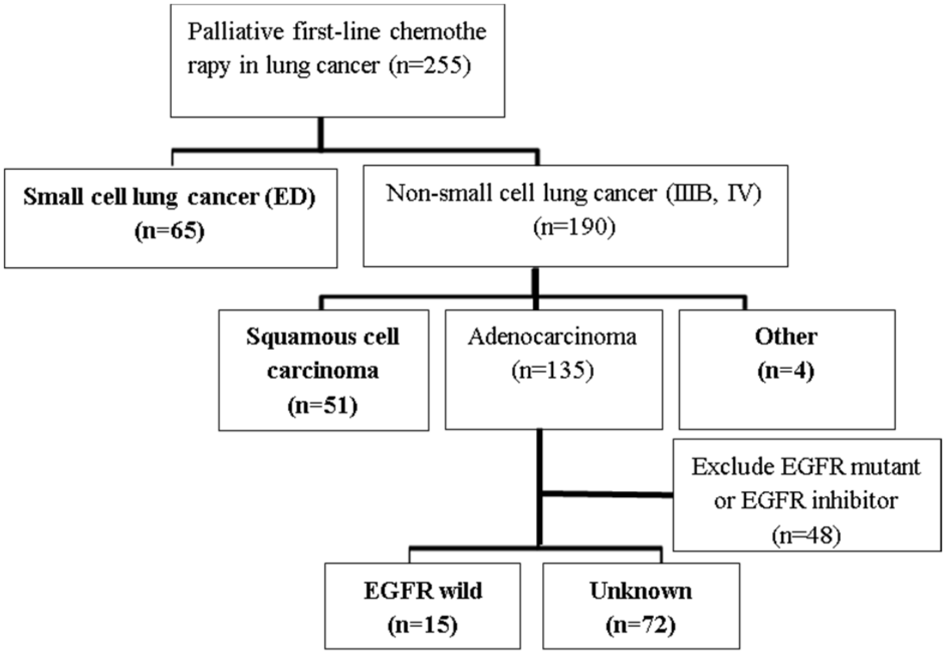

Supplement: Supplementary file 1 — Supplementary figure and table. [file jcav10p5332s1.pdf]
